# Draft Genome Sequence of Riemerella anatipestifer Isolate 17CS0503

**DOI:** 10.1128/genomeA.00274-18

**Published:** 2018-05-17

**Authors:** Anne Busch, Martin Ryll, Alexander Immel, Sabin Kornell, Ben Krause-Kyora, Herbert Tomaso, Helmut Hotzel

**Affiliations:** a Institute of Bacterial Infections and Zoonoses at the Friedrich-Loeffler-Institut, Federal Research Institute for Animal Health, Jena, Germany; b Stiftung Tierärztliche Hochschule Hannover, Hannover, Germany; c Institute of Clinical Molecular Biology, Kiel University, Kiel, Germany

## Abstract

Riemerella anatipestifer is a Gram-negative bacterium belonging to the family Flavobacteriaceae. It is primarily associated with acute septicemia in younger birds. The R. anatipestifer isolate 17CS0503 described here was isolated from a Peking duck (Anas platyrhynchos domesticus) in Hannover, Germany, in 1999.

## GENOME ANNOUNCEMENT

Riemerella anatipestifer is a Gram-negative bacterium belonging to the family Flavobacteriaceae. It is associated with acute septicemia in younger birds and is distributed worldwide in birds, thus being of major economic significance (1–7).

The isolate R. anatipestifer 17CS0503 was isolated from a Peking duck (Anas platyrhynchos domesticus) in Hannover, Germany, in 1999. The duck showed clinical signs of sinusitis, and the microorganism was cultivated from *sinus infraorbitalis* on blood agar with incubation in an atmosphere of 10% CO_2_ for 48 h at 37°C. DNA for whole-genome sequencing was prepared from a 2-ml culture in Mueller-Hinton broth (72 h at 37°C). DNA was purified using the High Pure PCR template preparation kit (Roche Diagnostics, Mannheim, Germany). The sequencing library was generated using the Nextera XT DNA library prep kit (Illumina, Inc., San Diego, CA, USA). From an Illumina HiSeq run with an average read length of 150 bp and an expected insert size of 350 bp, 18 million paired-end reads were generated (mean sequencing depth, >16,000 reads). Further read processing included quality trimming and assembly with SPAdes 3.9.1 in Bayes-Hammer mode (8), excluding contamination by removing contigs with a coverage of <30 and a length of <500 and assignment of the taxonomic labels to all contigs with Kraken version 0.10.6 (9). Annotation was performed with Prokka with the recommended standard settings (10). The final genome assembly was represented by 31 contigs with 2,127,386 bp. The G+C content was 35.1%. Annotation features include 1,960 genes, 3 rRNAs, 36 tRNAs, 2 repeat regions, and one transfer-messenger RNA (tmRNA). Analyses based on sequence identity with RAST (11) revealed the closest relationship to be with R. anatipestifer DSM 15868, isolated from Hannover, Germany (12).

R. anatipestifer is known to have a wide variety of resistances to antibiotics, and the identification of acquired antimicrobial resistance genes was performed (13). Resistance to tetracycline for isolate 17CS0503 was suspected by the presence of the *tet*(X) gene, detected in NODE_19 with a length of 2,287 bp. It had 95.03% identity to the *tet*(X) locus GU014535. Isolate 17CS0503 was tested *in vivo* for the MIC(s) of different antibiotics with Etest (bioMérieux, Nürtingen, Germany), according to the recommendations of the manufacturer. No resistance to erythromycin, doxycycline, tetracycline, streptomycin, gentamicin, chloramphenicol, ciprofloxacin, or teicoplanin could be detected. Because no interpretative criteria of MIC values are available for R. anatipestifer, the criteria for Escherichia coli were used (14). Even though *in silico* putative tetracycline resistance was predicted, no such resistance could be detected *in vivo*.

To test the evolution of antibiotic resistance within R. anatipestifer, 50,000 reads from metagenomic shotgun libraries from 1,400-year-old remnants of a rat found in a Roman settlement in Serbia (15) were mapped on the sequence of R. anatipestifer isolate 17CS0503. Analysis showed preferential mapping in a GT and a CCTT repeat region, and, unfortunately, no further analysis could be performed.

### Accession number(s).

This whole-genome shotgun project for Riemerella anatipestifer 17CS0503 has been deposited at GenBank under the accession number PKKR00000000, BioProject PRJNA423179, and BioSample SAMN08213267.
